# Integrative metagenomic and metabolomic analyses reveal gut microbiota-derived multiple hits connected to development of gestational diabetes mellitus in humans

**DOI:** 10.1080/19490976.2022.2154552

**Published:** 2022-12-22

**Authors:** Dewei Ye, Jiating Huang, Jiaming Wu, Kang Xie, Xiang Gao, Kaixuan Yan, Pengfei Zhang, Ying Tao, Yun Li, Shufei Zang, Xianglu Rong, Jun Li, Jiao Guo

**Affiliations:** a Key Laboratory of Glucolipid Metabolic Diseases of the Ministry of Education, Guangdong Pharmaceutical University, Guangzhou, China; b Guangdong Metabolic Disease Research Center of Integrated Chinese and Western Medicine, Guangdong Pharmaceutical University, Guangzhou, China; c Guangdong TCM Key Laboratory for Metabolic Diseases, Guangdong Pharmaceutical University, Guangzhou, China; d Key Laboratory of Metabolic Phenotyping in Model Animals, Guangdong Pharmaceutical University, Guangzhou, China; e Shenzhen Research Institute, City University of Hong Kong, Shenzhen, China; f The First Affiliated Hospital/School of Clinical Medicine, Guangdong Pharmaceutical University, Guangzhou, China; g Department of Endocrinology, Shanghai Fifth People’s Hospital, Fudan University, Shanghai, China; h Department of Infectious Diseases and Public Health, Jockey Club College of Veterinary Medicine and Life Sciences, City University of Hong Kong, Hong Kong, China; i School of Data Science, City University of Hong Kong, Hong Kong, China

**Keywords:** Glucose intolerance, insulin resistance, microbial metabolite, metabolic inflammation

## Abstract

Gestational diabetes mellitus (GDM) is characterized by the development of hyperglycemia and insulin resistance during the second or third trimester of pregnancy, associated with considerable risks to both the mother and developing fetus. Although emerging evidence suggests an association between the altered gut microbiota and GDM, remarkably little is known about the microbial and metabolic mechanisms that link the dysbiosis of the gut microbiota to the development of GDM. In this study, a metagenome-wide association study and serum metabolomics profiling were performed in a cohort of pregnant women with GDM and pregnant women with normal glucose tolerance (NGT). We identified gut microbial alterations associated with GDM and linked to the changes in circulating metabolites. Blood metabolite profiles revealed that GDM patients exhibited a marked increase in 2-hydroxybutyric acid and L-alpha-aminobutyric acid, but a decrease in methionine sulfoxide, allantoin, and dopamine and dopaminergic synapse, when compared with those in NGT controls. Short-chain fatty acid-producing genera, including *Faecalibacterium, Prevotella*, and *Streptococcus*, and species *Bacteroides coprophilus, Eubacterium siraeum, Faecalibacterium prausnitzii, Prevotella copri*, and *Prevotella stercorea*, were significantly reduced in GDM patients relative to those in NGT controls. Bacterial co-occurrence network analysis revealed that pro-inflammatory bacteria were over-represented as the core species in GDM patients. These microbial and metabolic signatures are closely associated with clinical parameters of glucose metabolism in GDM patients and NGT controls. In conclusion, we identified circulating dopamine insufficiency, imbalanced production of SCFAs, and excessive metabolic inflammation as gut microbiota-driven multiple parallel hits linked to GDM development. This work might explain in part the mechanistic link between altered gut microbiota and GDM pathogenesis, and suggest that gut microbiota may serve as a promising target to intervene in GDM.

## Introduction

Gestational diabetes mellitus (GDM) is characterized by glucose intolerance with onset or first recognition during pregnancy. It affects 3–14% of pregnant women worldwide^1^ and is associated with significant risks for maternal and fetal outcomes during pregnancy, delivery, and beyond. Women with GDM are at increased risk of pre-eclampsia, birth injury, and the need for operative delivery.^2^ Long-term maternal risks include GDM recurrence, metabolic syndrome, cardiovascular disease, and an increased incidence of antenatal or postpartum depression.^3^ Maternal hyperglycemia during pregnancy is also closely related to multiple neonatal, infant, and later outcomes in offspring, including large-for-gestational-age and fetal macrosomia. These outcomes are largely a result of the intrauterine environment with increased concentration of glucose and subsequent enhanced insulin secretion in the fetus, increased risk of injury during birth, and susceptibility to markedly increased adiposity during infancy and obesity-related long-term morbidities.^4^ Epidemiological studies have identified several risk factors for GDM such as advanced maternal age, maternal overweight and obesity, ethnicity, previous history of GDM, and family history of type 2 diabetes mellitus. Nonetheless, optimal management of the long-term outcomes of GDM in the mother and infant remains challenging,^5^ primarily because the molecular mechanisms underlying GDM pathogenesis have been poorly characterized.

Mounting evidence suggests that the gut microbiota plays a crucial role in the homeostatic regulation of glucose metabolism.^6^ Dysbiosis of the gut microbiota mediates the development of glucose intolerance and insulin resistance in type 2 diabetes mellitus through multiple mechanisms, including low-grade endotoxemia as a result of enhanced gut permeability, imbalanced generation of short-chain fatty acids (SCFAs) and branched-chain amino acids, and disturbed bile acid metabolism.^7^ Data from type 2 diabetes mellitus studies reveal that the gut microbiome may be a therapeutic target for glycemic control and function as a key mediator for the therapeutic benefits of anti-diabetic drugs.^8^ Epidemiological data have established a strong association between type 2 diabetes mellitus and GDM.^9^ Pathophysiologically, GDM shares several features with type 2 diabetes mellitus, including pancreatic β-cell dysfunction, and insulin resistance in parallel with defective insulin actions.^5^ Nonetheless, the relationship between GDM and gut microbiome is poorly characterized.

Pregnancy is associated with an adaptive response to hormonal and metabolic changes primarily driven by the feto-placental unit.^5^ As a master regulator to coordinate systemic glucose homeostasis, insulin signaling adapts to maintain normoglycemia in early pregnancy and ensures a sufficient supply of nutrients to the fetus, such as increased insulin secretion from pancreatic β-cells and blunted response to insulin-stimulated glucose uptake in peripheral tissues, especially skeletal muscle.^5^ Insulin resistance is an established trigger of hyperglycemia during pregnancy. Increasing evidence suggests that changes to the gut microbiome play a key role in mediating the development of insulin resistance in the presence of obesity and type 2 diabetes mellitus.^7^ Although several observational studies employing gut microbiome alone have shown that GDM patients exhibit evident changes in microbiome signature,^10–12^ the mechanistic links between altered gut microbiome and the development of GDM are not fully understood. Given that various circulating metabolites function as intermediaries between gut microbiome and host biology,^7,13^ the integrated analyses of microbial metabolites, gut microbiome, and host phenotype may represent a promising strategy to mine these high-dimensional data and yield mechanistic insight into the development of GDM and other multifactorial metabolic diseases.^14,15^

In the present study, we conducted integrative metagenomic and metabolomic analyses in a cohort of women with GDM and pregnant women with normal glucose tolerance (NGT) to identify potential mechanistic links between gut microbial composition and clinical phenotypes. Our metabolome–microbiome dual-omics analyses demonstrated neuroendocrine dysfunction characterized by dopamine insufficiency, excessive metabolic inflammation, and an imbalance in SCFAs as gut microbiota-driven multiple parallel hits linked to GDM development.

## Results

### Basic characteristics of the study cohort

**Figure 1.**
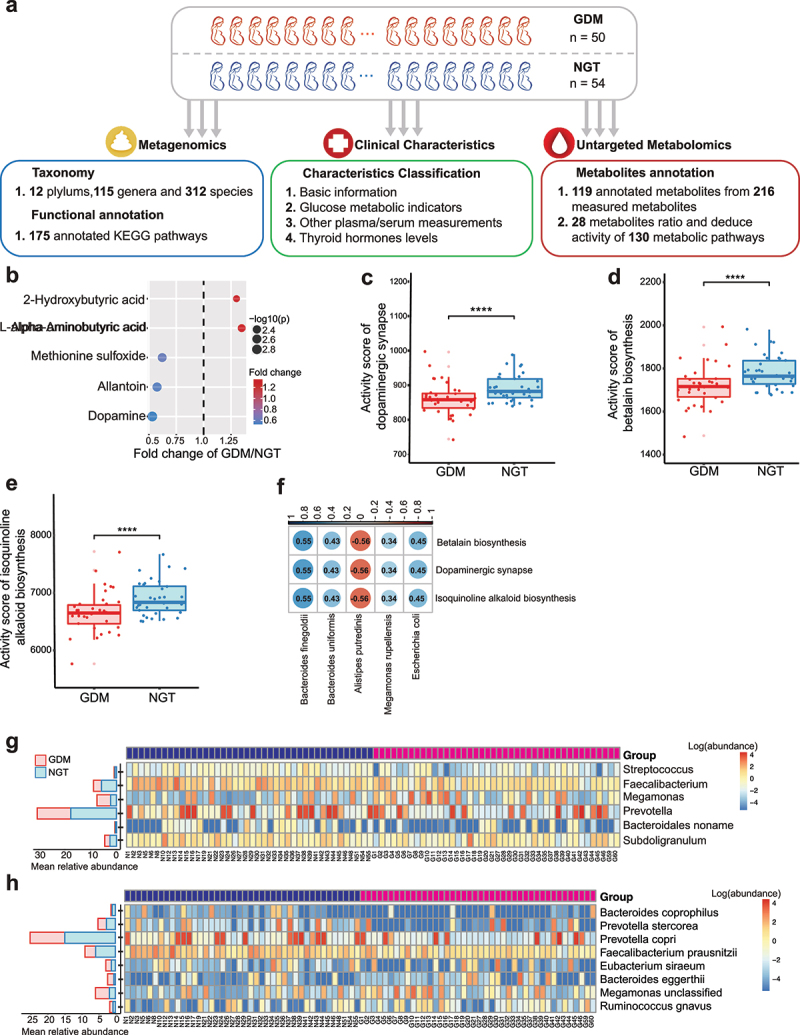
Alterations in gut microbiome and blood metabolites in GDM patients and pregnant women with NGT.

This study recruited 50 women with GDM and 54 healthy women with NGT (Figure 1a). Their characteristics are detailed in Supplementary Table 1. Age has not significant difference (*p* = .128, Wilcoxon rank-sum test) between GDM (31.74 ± 5.45, year) and NGT (30.20 ± 4.77, year) groups. GDM patients exhibited significantly higher pre-pregnancy BMI than their NGT counterparts (20.19 ± 3.90 *vs*. 17.11 ± 1.81 kg/m^2^, FDR *p* = .032, Wilcoxon rank-sum test), whereas BMI in the 24^th^ week of pregnancy showed no significant difference. GDM patients had significantly elevated levels of TG (2.32 ± 0.74 *vs*. 1.97 ± 0.63 mmol/L), neutrophil counts (7.89 ± 1.89 *vs*. 6.98 ± 1.95, 10^9^/L), and neutrophil percentage (76 ± 5 *vs*. 73 ± 5%) in blood relative to NGT controls (*p* = .039, *p* = .020, and *p* = .011, respectively, Wilcoxon rank-sum test). Additionally, GDM patients had significantly higher level of blood insulin (7.41 ± 11.52 *vs*. 2.86 ± 3.02, µIU/mL), HOMA-IR (1.53 ± 2.33 *vs*. 0.53 ± 0.58), and Hb1Ac (5.17 ± 0.44 *vs*. 5.00 ± 0.31%) compared with healthy controls (*p* = .026, *p* = .017, and *p* = .034, respectively, Wilcoxon rank-sum test) that indicated the dysfunction of glucose metabolism in GDM patients.

(**a**) Workflow of integrative metagenomic and metabolomic analyses in this study. (**b**) Profiling of blood metabolites. Metabolites with a cutoff FDR *p* < .2 were selected and presented as a fold-change of value in patients with gestational diabetes mellitus (GDM) relative to those in pregnant women with normal glucose tolerance (NGT). The size of circles was proportional to the -log_10_(*p*) value. (**c–e**) PAPi score of metabolic pathways of dopaminergic synapse (**c**), betalain biosynthesis (d), and isoquinoline alkaloid biosynthesis e) in GDM patients and NGT controls. **** FDR *p* < .01. (**f**) Gut microbial species linked to significantly altered blood metabolic pathway (FDR *p* < .2). (**g, h**) Gut microbial genus (**g**) and species (**h**) with differentiated alterations between GDM patients and NGT controls (FDR *p* < .2).

### Alteration of plasma metabolites and gut microbiome in GDM patients

We next conducted metabolite profiling in serum samples from GDM and NGT subjects based on untargeted metabolomics. We identified 119 annotated metabolites from a total of 216 metabolites measured (Figure 1a). GDM patients exhibited a marked increase in 2-hydroxybutyric acid (2-HB) and l-alpha-aminobutyric acid but a significant decrease in methionine sulfoxide, allantoin, and dopamine (FDR *p* < .2, Wilcoxon rank-sum test, Figure 1b). Metabolic pathway analysis with pathway activity profiling (PAPi) revealed that multiple pathways connected to dopamine metabolism, including dopaminergic synapse, betalain biosynthesis, and isoquinoline alkaloid biosynthesis, exhibited substantially lower PAPi scores in the GDM group compared with NGT controls (FDR *p* < .2, Wilcoxon rank-sum test, Figure 1c-e). In addition, a Sparse Partial Least Squares (sPLS) regression analysis demonstrated that the abundance of *Bacteroides finegoldii, Bacteroides uniformis, Megamonas rupellensis, Escherichia coli* positively correlated with dopaminergic synapse, betalain biosynthesis, and isoquinoline alkaloid biosynthesis, respectively, while the abundance of *Alistipes putredinis* negatively correlated with these three dopamine-related pathways (Figure 1f). These results indicated the close association between impaired dopamine metabolism and GDM.

The beta diversity and alpha diversity of the gut microbiota were similar in GDM and NGT groups (*p* = .068 in the ANOSIM test), while the richness was markedly reduced in GDM patients (*p* = .028, Wilcoxon rank-sum test, Supplementary Fig. 1A-C). We further compared the gut microbial composition at phylum, genus, and species levels between two groups. At the phylum level, women with GDM exhibited more abundant *Verrucomicrobia* (FDR *p* ≤ .07, Wilcoxon rank-sum test, Supplementary Fig. 2). The abundance of genera *Faecalibacterium, Prevotella*, and *Streptococcus*, which are involved in the production of SCFAs through the fermentation of dietary fiber,^16^ was significantly decreased in the GDM group. In contrast, the abundance of genus *Megamonas* was significantly higher in the GDM group (FDR *p* < .2, Wilcoxon rank-sum test, Figure 1g). The increased *Prevotella* abundance resulted in a higher *Prevotella*/*Bacteroides* ratio, which is closely associated with dietary fiber-induced improvement in glucose metabolism.^17^ Our data demonstrated that the *Prevotella*/*Bacteroides* ratio in GDM patients was markedly lower than that in the NGT group (*p* < .01, Wilcoxon rank-sum test, Supplementary Fig. 3). At the species level, we observed markedly differentiated abundance of species corresponding to the production of SCFAs,^18^ evidenced by a substantial reduction in the abundance of *Bacteroides coprophilus, Eubacterium siraeum, Faecalibacterium prausnitzii, Prevotella copri*, and *Prevotella stercorea* in parallel with a significantly increased abundance of *Bacteroides eggerthii, Megamonas* unclassified and *Ruminococcus gnavus* in the GDM group (FDR *p* < .2, Wilcoxon rank-sum test, Figure 1h). Furthermore, among a total of 17 KEGG functional pathways with significantly enriched abundance in GDM patients (FDR *p* < .2, Wilcoxon rank-sum test, Supplementary Fig. 4), the metabolic pathways of histidine metabolism and beta-alanine metabolism have been reported to impact capacity for SCFA production.^18^ Hence, our results indicate that the markedly reduced abundance of specific gut microbes may contribute to decreased production of SCFAs in GDM patients.

### Distinct pattern of bacterial co-occurrence network in GDM

**Figure 2.**
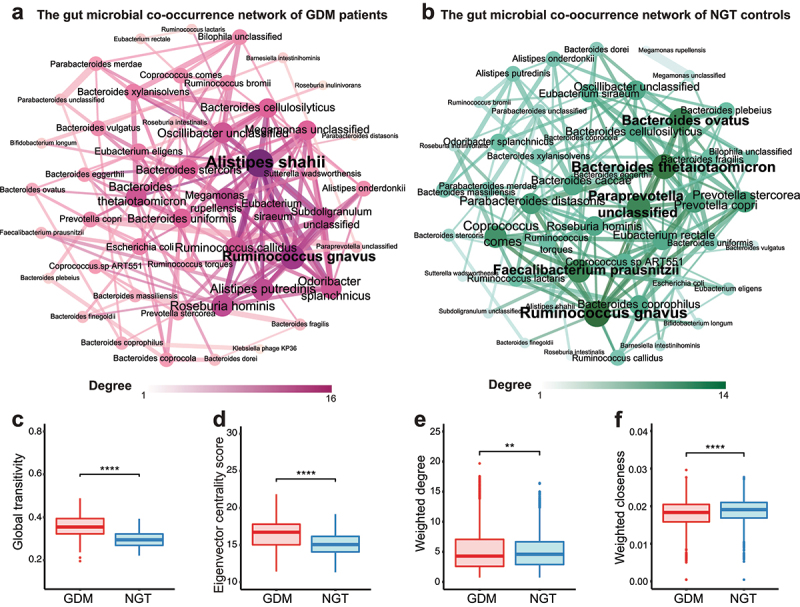
Analysis of gut bacterial co-occurrence network in GDM patients and pregnant women with NGT.

Since the co-occurrence of gut microbes reflects the interactions of microbes within an ecosystem, we conducted Spearman’s correlation analysis (FDR *p* < .05 and the correlation coefficient > |0.3| for the top 50 most abundant species) of bacterial species in GDM patients and NGT controls. We identified *Alistipes shahii* and *Ruminococcus gnavus* in women with GDM and *Bacteroides thetaiotaomicron, Faecalibacterium prausnitzii*, and *Ruminococcus gnavus* in NGT controls as the core species with the highest degree (Figure 2a-b). To further validate the different bacterial co-occurrence relationships between two groups, 30 randomly selected individuals from each group were used for network construction (permutation = 100 times). GDM patients displayed higher global transitivity (*p* < .0001, Wilcoxon rank-sum test) and eigenvector centrality score (*p* < .0001, Wilcoxon rank-sum test) but lower weighted degree (*p* < .01, Wilcoxon rank-sum test) and weighted closeness (*p* < .0001, Wilcoxon rank-sum test) relative to NGT controls (Figure 2c–f), indicating that hyperglycemia in GDM patients may blunt some beneficial interactions of the gut microbiota.

(**a, b**) Co-occurrence network of gut microbial species in patients with GDM (a) and in pregnant women with NGT (b). Node size represents the degree of one species in each network. Only the species connections (edges) > cutoff (correlation values > |0.3|, *p* < .05) are retained. Edge width represents the correlation value supporting the connection. (**c-f**) Global transitivity (c), eigenvector centrality score (d), weighted degree (e), and weighted closeness (f) of individual species in bootstrapped bacterial co-occurrence networks. A total of 30 samples were randomly selected and bootstrapped 100 times in either the GDM or NGT group to construct the bootstrapped networks.

**Figure 3.**
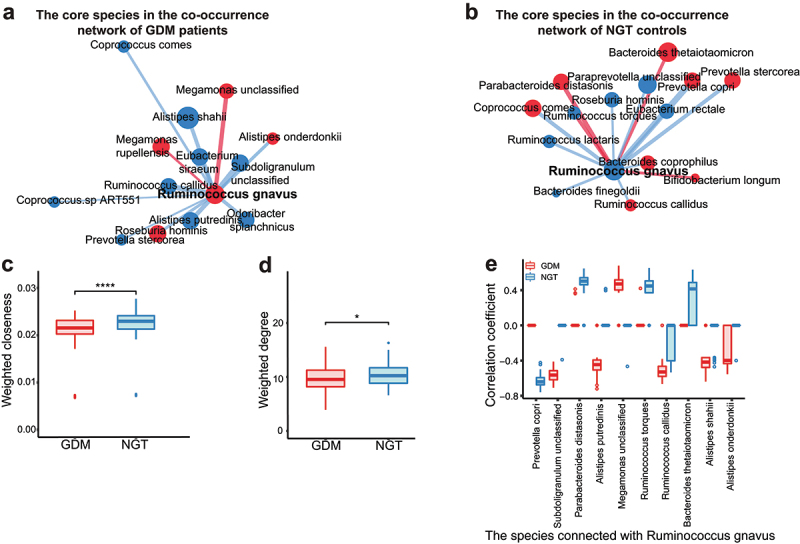
Analysis of core species in gut bacterial co-occurrence network in GDM patients and pregnant women with NGT.

To gain further mechanistic and functional insights into the core species of the bootstrapped co-occurrence networks in the development of insulin resistance and glucose intolerance in GDM, we ranked the weighted degree and weighted closeness of the species in networks from GDM and NGT, respectively. Intriguingly, several proinflammatory species, such as *Alistipes shahii*,^19^ *Ruminococcus gnavus*,^20^ *Ruminococcus callidus*,^21^ and *Alistipes putredinis*,^19^ were more likely to constitute core species of the gut microbiota in GDM patients (Supplementary Fig. 5A, C), whereas multiple species conferring the production of SCFAs, including *Bacteroides thetaiotaomicron*,^22^ *Prevotella copri*,^18^ *Faecalibacterium prausnitzii*,^18^ and *Bacteroides ovatus*^23^ were the core species in NGT patients (Supplementary Fig. 5B, D). Since *Ruminococcus gnavus* was identified as a core species in co-occurrence networks from both GDM and NGT patients, we investigated the differences in the connection of *Ruminococcus gnavus* between GDM and NGT groups (Figure 3a-b). *Ruminococcus gnavus* was positively associated with a *Megamonas* unclassified species in the GDM network (Figure 3a) and negatively associated with *Prevotella copri* and *Prevotella stercorea* in the NGT network (Figure 3b). Moreover, *Ruminococcus gnavus* showed significantly higher weighted degree and weighted closeness in the NGT bootstrap-constructed networks than in those for the GDM group (*p* < .05, Wilcoxon rank-sum test, Figure 3c, d). The distribution of correlation coefficients related to *Ruminococcus gnavus* was negatively correlated with the SCFAs-producing species *Prevotella copri*^18^ in NGT bootstrap-constructed networks (*p* < .05, Wilcoxon rank-sum test, Figure 3e). In contrast, *Ruminococcus gnavus* showed a positive correlation with a *Megamonas* unclassified species in GDM bootstrap-constructed networks (*p* < .05, Wilcoxon rank-sum test, Figure 3e), which was previously reported as a potential pro-inflammatory species.^24^ These data reveal different patterns in the bacterial co-occurrence networks between GDM patients and NGT controls. The core species in GDM networks tend toward pro-inflammatory species, while those in the NGT network tend toward SCFA-producing species.

(**a, b**) The connections of core species extracted from the original gut bacterial co-occurrence network of GDM (a) and NGT (b). (**c**) The weighted closeness of *Ruminococcus gnavus* in the 100 bootstrapped networks. (**d**) The weighted degree of *Ruminococcus gnavus* in the 100 bootstrapped networks. (**e**) The correlation values supporting the connection between *Ruminococcus gnavus* and other species in the 100 bootstrapped networks. The top 10 most significant differential distribution of correlation values (FDR *p* < .01) between GDM and NGT are shown in the plot. **** *p* < .0001, ** *p* < .01, * *p* < .05.

### Gut microbial signatures significantly correlate with the capacity of glucose tolerance during pregnancy

**Figure 4.**
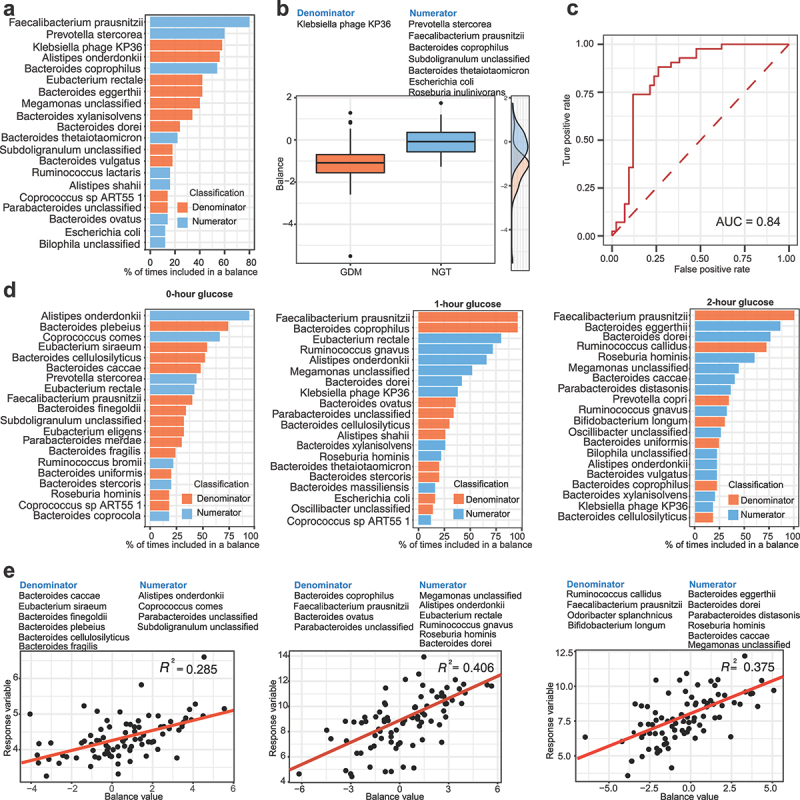
Association of gut microbial species with blood glucose level during oral glucose tolerance testing in GDM patients and pregnant women with NGT.

The balance between metabolically healthy microbiota and dysbiosis is crucial for maintaining host metabolic homeostasis^6^. Using ‘selbal’ package, we aimed to identify the balance of genera, the geometric means of relative abundances of several taxa associated with GDM. *Faecalibacterium prausnitzii* was the most frequent species (over 75%) in the balance of the cross validation between GDM and NGT groups (Figure 4a). Of note, GDM samples exhibited lower balance scores (Figure 4b). Furthermore, the balance showed a suitable apparent discrimination accuracy (AUC = 0.84, Figure 4c). To reveal the association of gut microbial balance with clinical biochemical parameters of glucose metabolism, the selbal algorithm was applied to search for a microbial signature that could predict glucose intolerance. *Alistipes onderdonkii* and *Bacteroides plebeius* were the top-ranked species related to basal glucose level during OGTT (Figure 4d, left). The analysis further demonstrated that *Faecalibacterium prausnitzii* was the most frequently observed species in the balance for blood glucose levels at 1- and 2-hours during OGTT (Figure 4d, middle and right panels). The ratio with lower abundance of species such as *Bacteroides cellulosilyticus* in the denominator to higher abundance of species such as *Alistipes onderdonkii* in the numerator was associated with fasting glucose levels (R^2^ = 0.285, Figure 4e, left). Consistently, the ratio of gut microbial species with lower abundance (such as *Bacteroides coprophilus* and *Faecalibacterium prausnitzii*) to gut microbial species with higher abundance (such as *Megamonas unclassified* and *Ruminococcus gnavus*) showed close association with 1-hour glucose level (R^2^ = 0.406, Figure 4e, middle). Likewise, the ratio of microbial species with lower abundance (such as *Ruminococcus callidus* and *Faecalibacterium prausnitzii*) to microbial species with high abundance (such as *Megamonas* unclassified and *Bacteroides eggerthii*) was associated with 2-hour glucose level during OGTT (R^2^ = 0.375, Figure 4e, right). Hence, the balance of specific microbial species was associated with clinical parameters of glucose tolerance during the second trimester of pregnancy.

(**a**) The frequency of gut microbial species selected in the balance of cross-validation process in association with GDM. The top-ranked 20 gut microbial species with the highest frequency are shown in the plot. Gut microbial species included in the numerator and the denominator of the balance are presented with blue color and orange color, respectively. (**b**) The two groups of gut microbial signature defining the global balance for GDM. (**c**) The apparent AUC value of the balance. (**d**) The frequency of gut microbial species selected in the balance of cross-validation process according to the association with blood glucose level at starting point (left panel), 1-hour (middle panel), and 2-hour (right panel) post-glucose administration during oral glucose tolerance testing (OGTT). (**e**) Association of the balance score (x-axis) with blood glucose level (y-axis) at starting point (left panel), 1-hour (middle panel), and 2-hour (right panel) post-glucose administration during OGTT. R-square values were calculated in the regression model.

### Association of gut microbial species with host circulating metabolites and clinical traits

**Figure 5.**
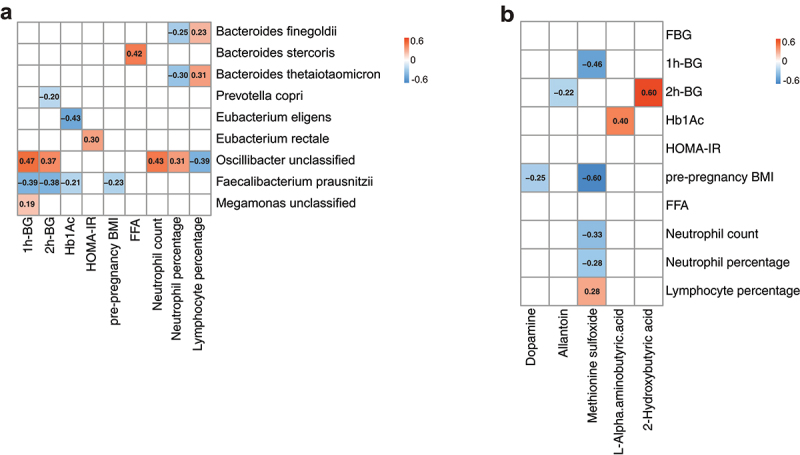
Association of representative gut microbial species and circulating metabolites with clinical indices.

Since pathological hyperglycemia as a result of glucose intolerance is the hallmark of GDM,^5^ screening of GDM is heavily dependent on OGTT at 24–28 weeks of gestation. In addition, prevailing evidence suggests that gut microbial metabolites function as a key mediator for the pleiotropic effects of gut microbiota on host metabolism.^7^ To delineate the pathophysiological relevance of GDM-related alterations to gut microbiota and its derived metabolites, we employed an sPLS regression model to analyze the association of key parameters of OGTT performed at 24–28 weeks of gestation with gut microbial species and circulating metabolites in GDM patients and NGT controls. The abundance of *Faecalibacterium prausnitzii* showed a significant and negative association with a cluster of clinical parameters including 1-hour glucose level in OGTT (correlation coefficient −0.39), 2-hour glucose level in OGTT (correlation coefficient −0.38), pre-pregnancy BMI (correlation coefficient −0.23), and Hb1Ac (correlation coefficient −0.21). The relative abundance of one *Oscillibacter* unclassified species was positively correlated with 1-hour glucose level in OGTT (correlation coefficient 0.47), 2-hour glucose level in OGTT (correlation coefficient 0.37), blood neutrophil count (correlation coefficient 0.43), and neutrophil percentage (correlation coefficient 0.31). Besides, *Bacteroides finegoldii* and *Bacteroides thetaiotaomicron* showed a positive correlation with blood lymphocyte percentage (correlation coefficient 0.23 and 0.31, respectively) but a negative correlation with neutrophil percentage (correlation coefficient −0.25 and −0.30, respectively). *Eubacterium rectale* had a positive correlation with HOMA-IR (correlation coefficient 0.30) (Figure 5a). Correlation analysis between gut microbiota and circulating metabolites revealed a significantly negative correlation for the abundance of *Bacteroides cellulosilyticus* with circulating 2-HB (correlation coefficient −0.37) and a positive correlation for the abundance of *Faecalibacterium prausnitzii* with circulating allantoin (correlation coefficient 0.49). Correlation analysis between metabolites and clinical phenotypes further demonstrated that blood 2-HB level was positively correlated with 2-hour blood glucose value (correlation coefficient 0.60) after high glucose administration in OGTT. The level of l-alpha-aminobutyric acid exhibited a positive correlation with blood Hb1Ac level. Additionally, the level of methionine sulfoxide was negatively correlated with pre-pregnancy BMI, blood neutrophil count, and neutrophil percentage (Figure 5b). Since neutrophils and innate immunity are emerging as important coordinators of metabolic and immune adaptations during pregnancy and circulating neutrophil count during the first-trimester is elevated and closely associated with the development of GDM in humans,^25^ our data suggest that GDM-related alterations of gut microbial species may link the neutrophil-mediated innate immunity and disorders in glucose metabolism during GDM development.

(**a**) Heatmap of Spearman’s correlation coefficient between the clinical indices with significant differences between GDM and NGT group (*p* < .05) and the GDM-associated gut microbial species. (**b**) Heatmap showing Spearman’s correlation coefficient between seven clinical indices with significant differences between GDM and NGT group (*P* < .05) and five circulating metabolites with significant alterations in GDM patients relative to NGT control subjects. 1 h-BG, blood glucose level at 1-hour post-glucose administration during OGTT; 2 h-BG, blood glucose level at 2-hour post-glucose administration during OGTT; FFAs, free fatty acids; Hb1Ac, hemoglobin A1c.

### GDM-associated gut microbial alteration links disordered dopamine metabolism

**Figure 6.**
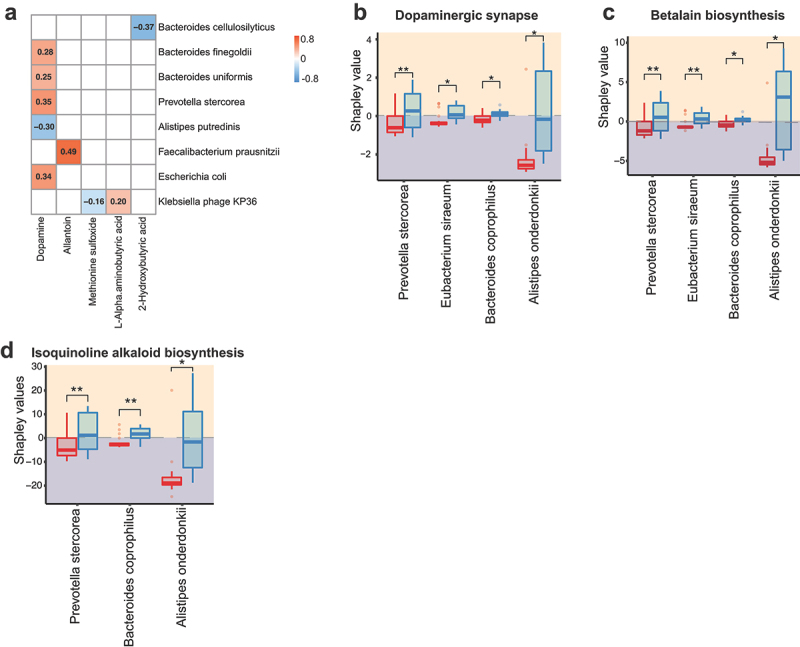
Association of gut microbial species with circulating metabolites.

SPLS analysis of gut microbiota with circulating metabolites revealed positive correlations for blood dopamine with the abundance of *Bacteroides finegoldii, Bacteroides uniformis, Prevotella stercorea*, and *Escherichia coli*, but a negative correlation with the abundance of *Alistipes putredinis* (Figure 6a). To further delineate the link between GDM-related gut microbial alteration and signals in dopamine metabolism, we calculated the species Shapley values in Random Forest (RF) regression model. Intriguingly, the significantly lower abundance of *Alistipes onderdonkii, Bacteroides coprophilus*, and *Prevotella stercorea* in the GDM group negatively contributed to the metabolic pathways involved in dopaminergic synapse (Figure 6b), betalain biosynthesis (Figure 6c), and isoquinoline alkaloid biosynthesis (Figure 6d) (FDR *p* < .05, Wilcoxon rank-sum test), all of which function as the key events in the cascade of dopamine metabolism (https://www.genome.jp/entry/map04728). These data that show a link between marked alteration in the abundance of gut microbial species and key steps in dopamine metabolism may provide further evidence of a close association between gut microbiota-driven disordered dopamine metabolism and GDM development.

(**a**) Spearman’s correlation of gut microbial species with circulating metabolites with significant alterations determined by cutoff FDR *p* < .2. (**b-d**) Shapley values of gut microbial species with significant changes between GDM and NGT groups in association with metabolic signals involved dopaminergic synapse (b), betalain biosynthesis (c), isoquinoline alkaloid biosynthesis (d). * FDR *p* < .05, ** FDR *p* < .01, *** FDR *p* < .001, **** FDR *p* < .0001.

## Discussion

Several previous reports employing only a metagenomic strategy demonstrated an association between altered gut microbial composition and clinical traits of GDM in humans.^10−12^ Nonetheless, the unique mechanism corresponding to the involvement of gut microbiome and metabolites in the development of worsened insulin resistance and glucose intolerance during GDM development remains poorly characterized. Given that microbial metabolites function as one of the primary modes by which the gut microbiota exerts important and diverse effects on host physiology,^7,13^ we employed integrative analyses of metagenomic sequencing and blood metabolites to explore the functional repertoire of complex bacterial communities in GDM. The dual-omics data in this study identified several parallel gut microbiota-driven alterations of immunometabolism in GDM patients, including dopamine insufficiency indicating neuroendocrine dysfunction, excessive metabolic inflammation, and an imbalance in the production of SCFAs. Our findings support the concept that the GDM-associated gut microbiome might prime neuroendocrine dysfunction, metabolic inflammation, and SCFA imbalance as multiple parallel hits, thereby contributing to GDM development.

Neuroendocrine control plays an important role in coordinating the homeostasis of glucose metabolism in multiple ways, including the regulation of hepatic glucose flux, control of glucose-stimulated insulin secretion, and counter-regulatory responses triggered by brain glucose sensors.^26^ Among the large array of hormones released from the nervous system, dopamine functions as an essential neuromodulator with pleiotropic roles in homeostatic regulation, including glucose metabolism. Dopamine regulates whole-body metabolism by controlling food selection, food intake, satiety, and energy expenditure.^27^ Compelling evidence from humans and rodents demonstrates a marked reduction in dopamine concentration and turnover in the hypothalamus, as well as lower dopamine D2 receptor (D2R) density in the striatum in the presence of obesity and type 2 diabetes mellitus,^27^ suggesting a strong link between dopamine insufficiency and obesity-induced diabetes. In contrast, optogenetic activation of dopamine D1 receptor-expressing neurons in the nucleus accumbens has been shown to promote glucose tolerance and insulin sensitivity in mice, highlighting dopamine-mediated striatal neuronal activity as an important regulator of systemic glucose metabolism.^28^ Pharmacologically, treatment with bromocriptine, a potent D2R agonist, improves glucose metabolism in obese subjects with insulin resistance.^29^ Dopamine in human amniotic fluid has been shown to be biologically active based on its ability to inhibit rat pituitary PRL secretion,^30^ suggesting a reciprocal relationship between dopamine and PRL in human amniotic fluid. Functionally, prolactin (PRL) regulates whole-body metabolic homeostasis through the modulation of key enzymes and transporters involved in glucose and lipid metabolism in adipose tissue and the pancreas.^31^ *In vitro* data demonstrate that PRL enhances the proliferation and insulin secretion in pancreatic β-cells via inhibition of cell apoptosis.^32^ Further, PRL attenuates fatty acid oxidation and enhances glucose-stimulated insulin secretion during pregnancy.^33^ The expression of PRL receptor in the liver is upregulated during pregnancy.^34^ Mice with genetic ablation of PRL receptor exhibit impaired glucose clearance, decreased glucose-stimulated insulin release, higher non-fasting blood glucose, and lower insulin levels during pregnancy.^35^ These data collectively may indicate that elevated circulating PRL acts as an adaptive response to maintain glucose tolerance during pregnancy. In this regard, our metabolomics data revealed that GDM patients demonstrated significantly decreased blood dopamine and a marked reduction in three important events involving dopamine metabolism (inducing dopaminergic synapse, betalain biosynthesis, and isoquinoline alkaloid biosynthesis). Furthermore, integrated analysis demonstrated a significant association between altered gut microbial species (*Bacteroides massiliensis, Bacteroides coprophilus*, and *Megamonas rupellensis*) and dopamine metabolism. Thus, our data demonstrate a link between pregnancy-related altered gut microbiome and dopamine insufficiency.

SCFAs, saturated fatty acids with a chain length ranging from one to six carbon atoms, are the main metabolites produced by bacterial fermentation of indigestible carbohydrates in the gastrointestinal tract. Butyrate, acetate, and propionate represent the main SCFAs produced by the gut microbiota.^18,36^ SCFAs are known to play pivotal roles in modulating the homeostasis of energy expenditure, immunity, and metabolism in the host through pleiotropic effects.^16^ Specifically, SCFAs regulate whole-body glucose metabolism in multiple ways including suppression of appetite by promoting the release of satiety hormones and stimulation of vagal afferent chemoreceptors; enhancement of energy expenditure by potentiating thermogenesis-related proteins in the liver and adipose tissue; and augmentation of glucose-stimulated insulin secretion from pancreatic β cells.^16^ Therapeutically, an overwhelming amount of animal data demonstrates the benefits of butyrate or propionate treatment on energy expenditure and glucose homeostasis.^7^ Consistently, human data demonstrate that enhancement of microbial production of SCFAs may function as an important mediator of the metabolic benefits of dietary fiber^16^ and the glucose-lowering drug metformin.^8^ This suggests that fermentation into SCFAs may represent not only an important mechanism underlying the involvement of gut microbiota in the development of glucose metabolic disorders but also an event that lends itself to therapeutic intervention. Although the dynamics of gut microbial SCFAs during pregnancy remain largely unknown, emerging data from cross-sectional studies reveal that SCFAs may play an important role in regulating glucose homeostasis during pregnancy.^37^ Thus, our data, together with a previous report demonstrating that *ex vivo* treatment with butyrate and propionate alleviates TNF- and lipopolysaccharide-induced release of pro-inflammatory cytokines and chemokines in human placenta, visceral adipose tissue, and subcutaneous adipose tissue,^38^ suggest that imbalanced gut microbial SCFAs may serve as an important pathogenic event during GDM development.

Metabolic inflammation that may be low-grade, chronic, or systemic in nature has been well established as an important player and a hallmark that contributes to the onset of insulin resistance and glucose intolerance during the development of obesity-induced type 2 diabetes mellitus.^39^ Obesity-evoked activation of innate immune modulators, including membrane-bound toll-like receptors and pyrin domain-containing protein 3 inflammasome, mediates the production of various proinflammatory cytokines (such as TNF-α, IL‑1β, and IL‑18) to trigger inflammatory responses in pancreatic islets, and adipose tissue, skeletal muscle, and liver tissue, thereby disrupting the homeostasis of glucose metabolism.^40^ Adipose tissue is a metabolically active endocrine organ that secretes a range of adipokines, cytokines and chemokines to regulate whole-body energy homeostasis. Compelling evidence suggests an inflammatory response in adipose tissue as the origin of sustained metabolic inflammation.^41^ The link between GDM and excessive adiposity during either pre-pregnancy or pregnancy is also well established, with emerging evidence of a close association between GDM and excessive and sub-clinical inflammation as a result of overactivation of innate immunity, mainly due to notable elevation of proinflammatory cytokines.^42^ Although the causative relationship between metabolic inflammation and insulin resistance during GDM development remains unclear, gut microbiota has been shown to be a driving force that fuels metabolic inflammation through the production of pro-inflammatory modulators or intestinal barrier dysfunction.^43^ Our analysis revealed altered gut macrobiotic composition and gut microbiota-derived metabolites in GDM patients. Together with previous reports,^16,20^ our data provide evidence that gut microbiota combined with its derived metabolites may function as a key contributor to GDM-related metabolic inflammation.

This study has limitations. First, gut microbiota is prone to regional and ethnic variations^44^ that may also apply in pregnant women and those with GDM. A principal limitation of the present study was its involvement of only Chinese subjects. Further validation in larger and more diverse populations is warranted. Second, evidence supporting the causal relationship between gut microbiome-derived “multiple hits” and GDM development is lacking due to the cross-sectional study design. The pathophysiological relevance and functional roles of gut microbiota-driven events in GDM development should be explored and confirmed by further studies employing human microbiota-associated rodents or other approaches with GDM-specific modification of gut microbiome.

**Figure 7.**
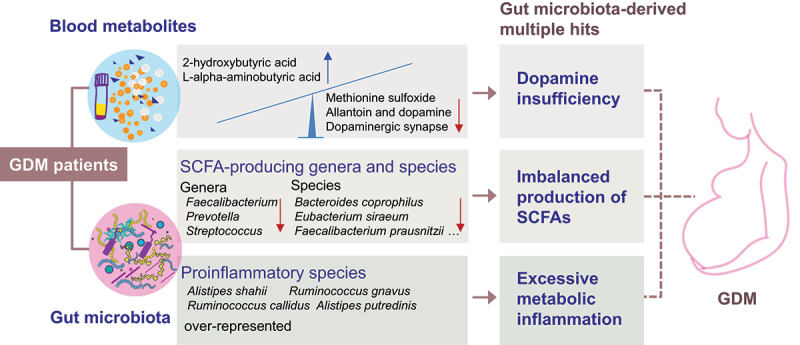
Schematic representation of mechanistic insights provided by the integrative metagenomic and metabolomic analyses in the present study of GDM development.

In conclusion, an integrative analysis employing metagenomic sequencing and bacterial metabolites in the present study identified dopamine insufficiency, an imbalance in the production of SCFAs, and excessive metabolic inflammation as gut microbiota-derived multiple parallel hits linked to the development of GDM in humans (Figure 7). These findings may provide a unified mechanism to explain how microbe–metabolite interactions contribute to GDM pathogenesis. Our data also indicate that gut microbiota and microbial metabolites may represent a promising target for novel therapeutic strategies to combat GDM.

Gut microbial dysbiosis coupled with gut microbial species with marked alteration may drive the substantial changes in circulating metabolites, thereby exerting profoundly deleterious effects on the homeostasis of glucose metabolism through multiple branches, including imbalance in the production of SCFAs, neuroendocrine dysfunction characterized by dopamine deficiency, excessive metabolic inflammation. Red arrow denotes the decreased level in GDM patients and blue arrow indicates the increased level in GDM patients.

## Patients and methods/materials

### Subjects and sample collection

Pregnant women were recruited from The First Affiliated Hospital, Guangdong Pharmaceutical University. All the women underwent a 75-g oral glucose tolerance test (OGTT) between 24 and 28 weeks of pregnancy. GDM was diagnosed when OGTT value fulfilled at least one of the following criteria: FPG ≥ 92 mg/dL, 1-h plasma glucose ≥180 mg/dL or 2-h plasma glucose ≥153 mg/dL.^45^ Pregnant women with NGT were defined as those with normal glucose levels on OGTT screening during the second trimester. Women were excluded if they had any of the following: diseases that might affect glucose metabolism or microbiome composition (pre-pregnancy diabetes, hypertension, thyroid disorders, asthma, lipid metabolic disorders, inflammatory bowel disease, irritable bowel syndrome, celiac disease); treatment with antibiotic(s) in the previous month; use of probiotics the last 2 weeks; smoking; heavy alcohol consumption (>120 g *per* week); family history of diabetes. A fasting blood sample was obtained prior to glucose intake and commencement of the OGTT and subsequently stored at −80°C. Fecal samples with stool DNA stabilizer were stored at −80°C until analysis. All participants provided written informed consent prior to collection of clinical information, fecal and blood samples. This study was approved by the Human Research Ethics Committee in The First Affiliated Hospital/School of Clinical Medicine, Guangdong Pharmaceutical University and carried out in accordance with the Helsinki Declaration.

### Metabolomic analysis of blood samples

The untargeted metabolomics profiling was performed on XploreMET platform (Metabo-Profile, China). Samples were prepared following previously reported procedures with minor modifications.^46^ Raw data generated by GC-TOF/MS were processed using XploreMET 3.0 (Metabo-Profile).

### Metagenomic sequencing, quality control, and microbial taxonomic profiling

Genomic DNA from fecal samples was extracted using the stool collection tubes in the PSP® Spin Stool DNA Plus Kit (Stratec, Cat No. 1038110300). DNA purity was checked by the Nano-Drop® spectrophotometer (Thermofisher). All samples were sequenced on the Illumina HiSeq 4000 platform (Illumina, San Diego, California, USA; Paired end; insert size, 350 bp; read length, 150 bp). The raw reads obtained from the sequencing platform were used for quality control. Human-derived reads were filtered by bwa men against the human reference genome that matched the cut-off: consecutive exact match ≥25 bp and identity ≥95%. Low-quality bases, reads, or PCR duplicates were removed as previously described.^47^ The script appears on GitHub (https://github.com/TingtZHENG/VirMiner/). After that, MetaphlAn2.0^48^ was used for taxonomic profiling of gut microbiome. A total of 212,199,828 high-quality paired sequences were classified into 12 taxa at phylums, 115 taxa at genus level, and 312 taxa at species level. Based on these profiles, the comparison of relative abundance and gut microbial diversity, co-occurrence network construction, and gut balance analysis are described in the *Supplementary Materials*.

### PAPi score prediction of metabolic pathway and Shapley values calculation

R package PAPi was used to predict PAPi score of metabolic pathways based on the abundance of the metabolites. Due to the unique capability of the Random Forest (RF) regression model to model nonlinear relationships and deal with multidimensional and multimodal features given a relatively small sample size (dozens to hundreds),^49^ we constructed an RF regression model based on the relative abundance of the top 50 most abundant species to predict the metabolic pathway with significantly different PAPi score. Shapley Additive Explanations (SHAP, R package *shapper*)^50^ were applied to calculate the Shapley value of the gut species in the RF regression model and explain the association of the gut species and metabolic pathways. The gut species with significantly different Shapley values was presented for each metabolic pathway. More details about the R packages used in this study are summarized in Supplementary Table 2.

### Assembly-free functional annotation of metagenomic data

Ten thousand high-quality reads were randomly subsampled from each sample and mapped to KEGG Orthologs (KO) database^51^ using blastx with an e-value cutoff of 1E-6. The identified KO categories were annotated according to KEGG pathways. The Wilcoxon rank-sum test and the Benjamini and Hochberg method were used to identify differentially abundant pathways with a false-discovery rate-corrected (FDR) p-value <0.2.

## Statistical analysis

The levels of the clinical phenotype, the abundance of metabolites, gut microbial species and genus, or KEGG functional pathway were determined by pairwise comparison with NGT controls using two-sided Wilcoxon rank-sum test. *P* < .05 was considered statistically significant. In addition, the FDR p-value was estimated for multiple comparisons using the Benjamini–Hochberg method.

## Supplementary Material

Supplemental Material

## Data Availability

The datasets generated during metagenomic sequencing of fecal DNA samples have been deposited in NCBI Sequencing Read Archive (accession ID: PRJNA853814). The R analysis scripts used in this paper have been deposited at GitHub at https://github.com/JiatingH/GDM-Project. Links to the algorithms applied in the study are listed in **Supplementary Table 2**. The information will be publicly available at the time of publication. Other datasets from the current study are available from the corresponding author upon request. Additional information of detailed methodology is described in ***Supplementary Materials*** section.

## Research highlights

- GDM patients exhibited marked changes in blood metabolites related to dopamine insufficiency.
- Short-chain fatty acid-producing gut microbial genera were substantially reduced, but pro-inflammatory bacteria were over-represented as the core species in GDM patients.
- Microbial and metabolic signatures are closely associated with clinical parameters of glucose metabolism in GDM patients and NGT controls.
- Dual-omics analyses in this study identified dopamine insufficiency, an imbalance in SCFAs, and excessive metabolic inflammation as gut microbiota-driven multiple parallel hits linked to GDM.
