# Supplementary material for: Integrative metagenomic and metabolomic analyses reveal gut microbiota-derived multiple hits connected to development of gestational diabetes mellitus in humans
Source: Gut Microbes. 2022 Dec 22;15(1):2154552. doi: 10.1080/19490976.2022.2154552 (PMC9794004; doi:10.1080/19490976.2022.2154552)
Supplement: Supplemental Material [file KGMI_A_2154552_SM9684.zip › Research highlights_20221102.docx]

**Research highlights**

- GDM patients exhibited marked changes in blood metabolites related to dopamine insufficiency.
- Short-chain fatty acid-producing gut microbial genera were substantially reduced, but proinflammatory bacteria were over-represented as the core species in GDM patients.
- Microbial and metabolic signatures are closely associated with clinical parameters of glucose metabolism in GDM patients and NGT controls.
- Dual-omics analyses in this study identified dopamine insufficiency, an imbalance in SCFAs, and excessive metabolic inflammation as gut microbiota-driven multiple parallel hits linked to GDM.
