# Supplementary material for: Integrative metagenomic and metabolomic analyses reveal gut microbiota-derived multiple hits connected to development of gestational diabetes mellitus in humans
Source: Gut Microbes. 2022 Dec 22;15(1):2154552. doi: 10.1080/19490976.2022.2154552 (PMC9794004; doi:10.1080/19490976.2022.2154552)
Supplement: Supplemental Material [file KGMI_A_2154552_SM9684.zip › Supplementary Material_20221102_1800_Final Version.pdf]

Supplementary Materials

For Manuscript titled **"Integrative Metagenomic and Metabolomic Analyses Reveal Gut Microbiota-Derived Multiple Hits Connected to Development of Gestational Diabetes Mellitus in Humans"** by Dewei Ye, *et al*.

1. Supplementary Methods

1.1. Gut microbial diversity and the comparison of the relative abundance

The alpha diversity was calculated using the Shannon index. The species richness was measured using Chao 1 estimator. For beta diversity analysis, the nonmetric multidimensional scaling (NMDS) plot was calculated based on Bray-Curtis dissimilarities matrix. ANOSIM test was used to determine the overall community composition difference between GDM and NGT samples. These calculations and visualization were implemented using the R package *vega*n. Due to the high heterogeneity and sparsity (tremendous amount of zeros in the profile) of the metagenomic profile, the species (or genus) of the 50 (or 25) that were most abundant in all the samples was screened for differential abundance analysis. Wilcoxon rank-sum tests were performed to detect the differentially abundant species and genus between GDM and NGT groups, followed by adjustment for multiple testing using the Benjamini and Hochberg method. Significantly different species (or genus) were identified with a false-discovery rate (FDR) p-value < 0.2.

1.2. Co-occurrence network construction and gut microbial balance analysis

Spearman's correlation analysis was used to reveal the taxonomic relationship at species level. The 50 most abundant species were included in the correlation analysis. A linkage with correlation coefficient > |0.3| in Spearman's correlation analysis and significance value < 0.05 was selected to construct the networks in GDM and NGT groups. Gephi was applied to visualize the network. Random selection of 30 species in the GDM and NGT cohorts was repeated 100 times to construct the sub-networks. The R package *igraph* was adopted to evaluate the structure of GDM and NGT sub-networks. Wilcoxon rank-sum test was performed to detect the structural difference between two groups with statistical

significance set at $p < 0.05$. The R package *selbal* was applied to explore the interaction of a group taxa (gut microbial balance) between GDM and NGT groups. The function 'selbal.cv' in the *selbal* package was used with the parameter 'user_numVar' set as 10.

1.3. Sparse Partial Least Squares regression

Sparse Partial Least Squares regression (sPLS) [1] was used to identify the correlation between clinical phenotype, gut species, and plasma metabolites. Significantly changed clinical phenotype ($p < 0.05$), plasma metabolites (FDR $p < 0.2$), and top 50 abundant species were selected for sPLS analysis. sPLS was performed using R package *spls*. The correlation coefficient was corrected using the function 'correct.spls'. After correction, the results were presented in the heatmap.

2. Supplementary Table

2.1. Supplementary Table 1. Clinical characteristics

	NGT	GDM	*p* value
Age (year)	30.20 (4.77)	31.74 (5.45)	0.128
Nulliparous/ multiparous	23/31	14/36	0.178
24W-WC (cm)	87.17 (6.51)	86.64 (9.01)	0.734
Pre-pregnant BMI (kg/m^2)	17.11 (1.81)	20.19 (3.90)	0.032*
24W-BMI (kg/m^2)	24.55 (3.15)	25.76(3.87)	0.082
FBG (mmol/L)	4.20 (0.38)	4.55(0.72)	0.002**
1h-BG during OGTT (mmol/L)	7.20 (1.59)	10.43 (1.23)	<0.001***
2h-BG during OGTT (mmol/L)	6.46 (1.10)	8.93(1.34)	<0.001***
Insulin (μIU/mL)	2.86 (3.02)	7.41 (11.52)	0.026
HOMA-IR index	0.53 (0.58)	1.53 (2.33)	0.017
Hb1Ac (%)	5.00 (0.31)	5.17 (0.44)	0.034*
WBC count (10^9/L)	9.48 (2.46)	10.28 (2.16)	0.087
Neutrophil percentage (%)	73 (5)	76 (5)	0.011*
Lymphocyte percentage (%)	20 (4)	17 (5)	0.014*
Mononuclear cell percentage (%)	5 (1)	7 (12)	0.164
Eosinophil percentage (%)	2 (1)	2 (2)	0.818
Basophils percentage (%)	0.3 (0.1)	0.2 (0.1)	0.406
Neutrophil count (10^9/L)	6.98 (1.95)	7.89 (1.89)	0.020*
Lymphocyte count (10^9/L)	1.81 (0.39)	1.75 (0.40)	0.487
Monocyte count (10^9/L)	0.47 (0.12)	0.51 (0.18)	0.171
Eosinophils count (10^9/L)	0.14 (0.10)	0.14 (0.08)	0.855
Basophils count (10^9/L)	0.03 (0.01)	0.03 (0.01)	0.763
TC (mmol/L)	5.70 (0.92)	5.45 (0.96)	0.266
TG (mmol/L)	1.97 (0.63)	2.32 (0.74)	0.039*
HDL (mmol/L)	1.96 (0.40)	1.85 (0.33)	0.242
TC/HDL ratio	2.98 (0.59)	3.00 (0.63)	0.915
LDL (mmol/L)	2.76 (0.83)	2.43 (1.00)	0.145
VLDL (mmol/L)	0.91 (0.31)	1.07 (0.36)	0.055
LDL/HDL ratio	1.59 (0.59)	1.47 (0.64)	0.405
ApoA1 (g/L)	2.09 (0.26)	2.15 (0.24)	0.332
Apo-B (g/L)	1.04 (0.21)	1.05 (0.24)	0.910
Non-HDL-C (mmol/L)	3.82 (0.87)	4.38 (3.39)	0.423

FFA (mmol/L)	0.36 (0.10)	0.46 (0.14)	0.003**
ALT (U/L)	17.76 (10.51)	18.39 (13.71)	0.809
AST (U/L)	20.13 (10.91)	17.95 (7.24)	0.283
TP (g/L)	65.40 (4.42)	65.02 (3.68)	0.671
Albumin (g/L)	37.96 (3.38)	37.27 (3.08)	0.329
Globulin (g/L)	27.44 (2.97)	28.10 (3.22)	0.331
Albumin/Globulin	1.40 (0.20)	1.61(1.54)	0.373
TBIL (umol/L)	8.04 (2.84)	7.83 (2.24）	0.717
DBIL (umol/L)	2.75 (1.23)	2.61(0.98)	0.587
IBIL (umol/L)	5.23 (2.10)	5.23 (1.72)	0.994
BUN (mmol/L)	2.94 (0.58)	2.88 (0.66)	0.637
Cre (umol/L)	44.62 (7.94)	45.27 (6.98)	0.691
Uric acid (umol/L)	263.52 (53.51)	267.70 (54.69)	0.725
Cystatin c (mg/L)	0.65 (0.09)	0.70 (0.11)	0.038*
SOD (U/mg)	124.78 (10.12)	124.12 (9.92)	0.779
TSH (μIU/mL)	1.84 (1.21)	1.71 (2.62)	0.758
FT4 (pmol/L)	15.43 (2.85)	14.01 (2.63)	0.021*
Anti-TPO (IU/mL)	18.82 (29.80)	21.47 (37.34)	0.752
FT3 (pmol/L)	4.84 (0.92)	4.71 (0.61)	0.591

Abbreviations: 24W-WC, 24th-week waist circumference; Pre-pregnant BMI, pre-pregnant body mass index; 24W-BMI,24th-week body mass index; FBG, fasting blood glucose; 1h-BG during OGTT, 1-hour blood glucose during oral glucose tolerance test；2h-BG, 2-hour blood glucose during oral glucose tolerance test; HOMA-IR index, homeostasis model assessment-insulin resistance index; Hb1Ac, glycosylated hemoglobin type A1C; WBC count, white blood cell count; TC, total cholesterol; TG, triglyceride; HDL, high density lipoprotein; LDL, low density lipoprotein; VLDL, very low-density lipoprotein；ApoA 1, apolipoprotein A1; ApoB, apolipoprotein B; non-HDL-C, non-high-density lipoprotein cholesterol; FFA, free fatty acid; ALT, alanine aminotransferase; AST, aspartate aminotransferase; TP, total protein; TBIL, total bilirubin; DBIL, direct bilirubin; IBIL, indirect bilirubin, BUN, blood urea nitrogen; Cre, creatinine; SOD, superoxide dismutase; TSH, thyroid stimulating hormone; FT4, free thyroxine; anti-TPO, thyroid peroxidase antibody; FT3, free triiodothyronine. The continuous variables were presented as Mean (Standard deviation). * $p < 0.05$, ** $p < 0.01$, *** $p < 0.001$.

2.2 Supplementary Table 2. Summary of algorithms applied in the study

Package	Version	Parameter setting	Links	Reference
vegan	2.5-7	Default	https://github.com/vegandevs/vegan/releases	[2]
selbal	0.1	user_numVar = 10	https://github.com/malucalle/selbal	[3]
igraph	1.3.5	Default	https://cran.r-project.org/web/packages/igraph/index.html	[4]
spls	2.2-3	K = 12 or 6, eta = 0.8	https://cran.r-project.org/web/packages/spls/index.html	[5]
PAPi	1.26.0	Default	https://www.bioconductor.org/packages//2.12/bioc/html/PAPi.html	[6]
shapper	0.1.3	Default	https://cran.r-project.org/web/packages/shapper/index.html	[7]

3. Supplementary Figures

3.1. Supplementary Fig. 1

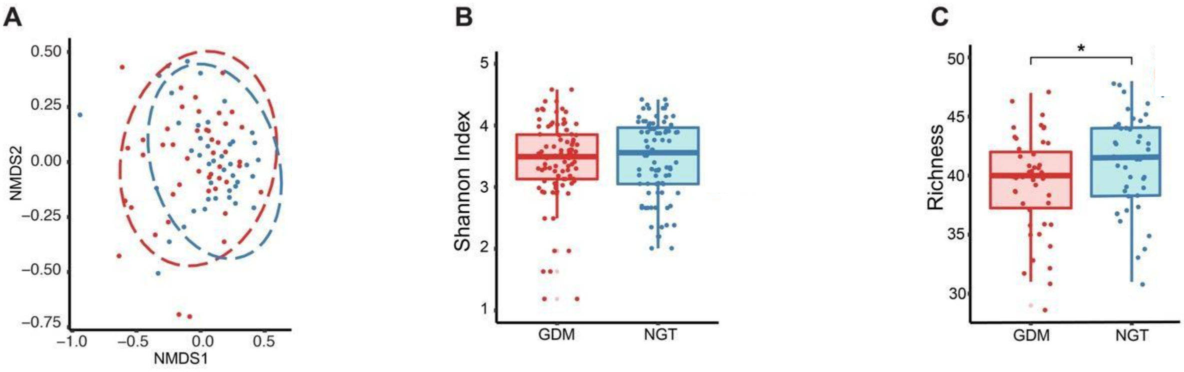

Supplementary Fig. 1 Beta diversity and alpha diversity of gut microbiome

(A) The nonmetric multidimensional scaling determined according to gut microbial species data. (B) Shannon index of gut microbiota. (C) Gut microbial richness valuated by the Chao 1 index. * $p < 0.05$.

3.2. Supplementary Fig. 2

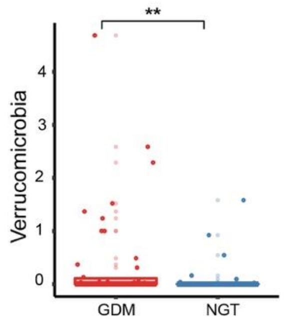

Supplementary Fig. 2 Abundance of gut microbial phylum *Verrucomicrobia*.

Data was compared according to gut microbial phylum profile. ** FDR $p < 0.1$.

3.3. Supplementary Fig. 3

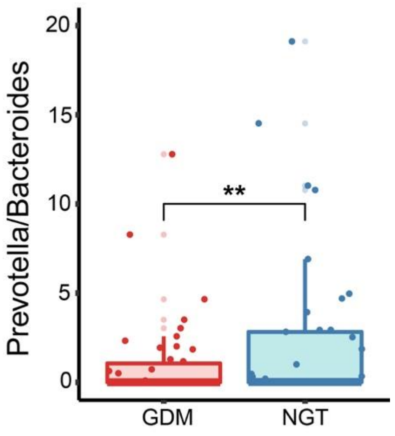

Supplementary Fig. 3. Decreased ratio of *Prevotella* to *Bacteroides* in GDM patients.

** $p < 0.01$.

3.4. Supplementary Fig. 4

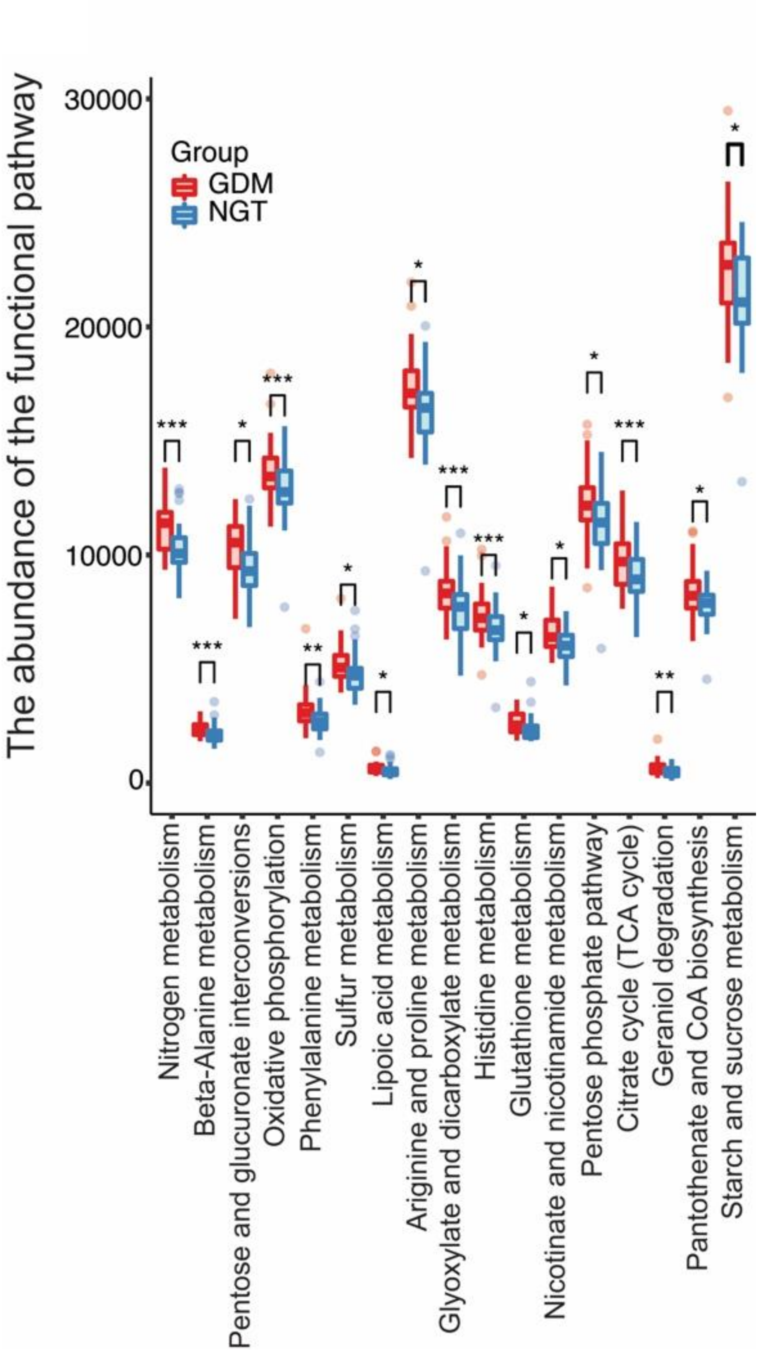

Supplementary Fig. 4. The abundance of the functional pathways.

Pathways with significant difference between GDM and NGT groups were presented. * FDR $p < 0.2$, ** FDR $p < 0.1$, *** FDR $p < 0.05$.

Supplementary Fig. 5

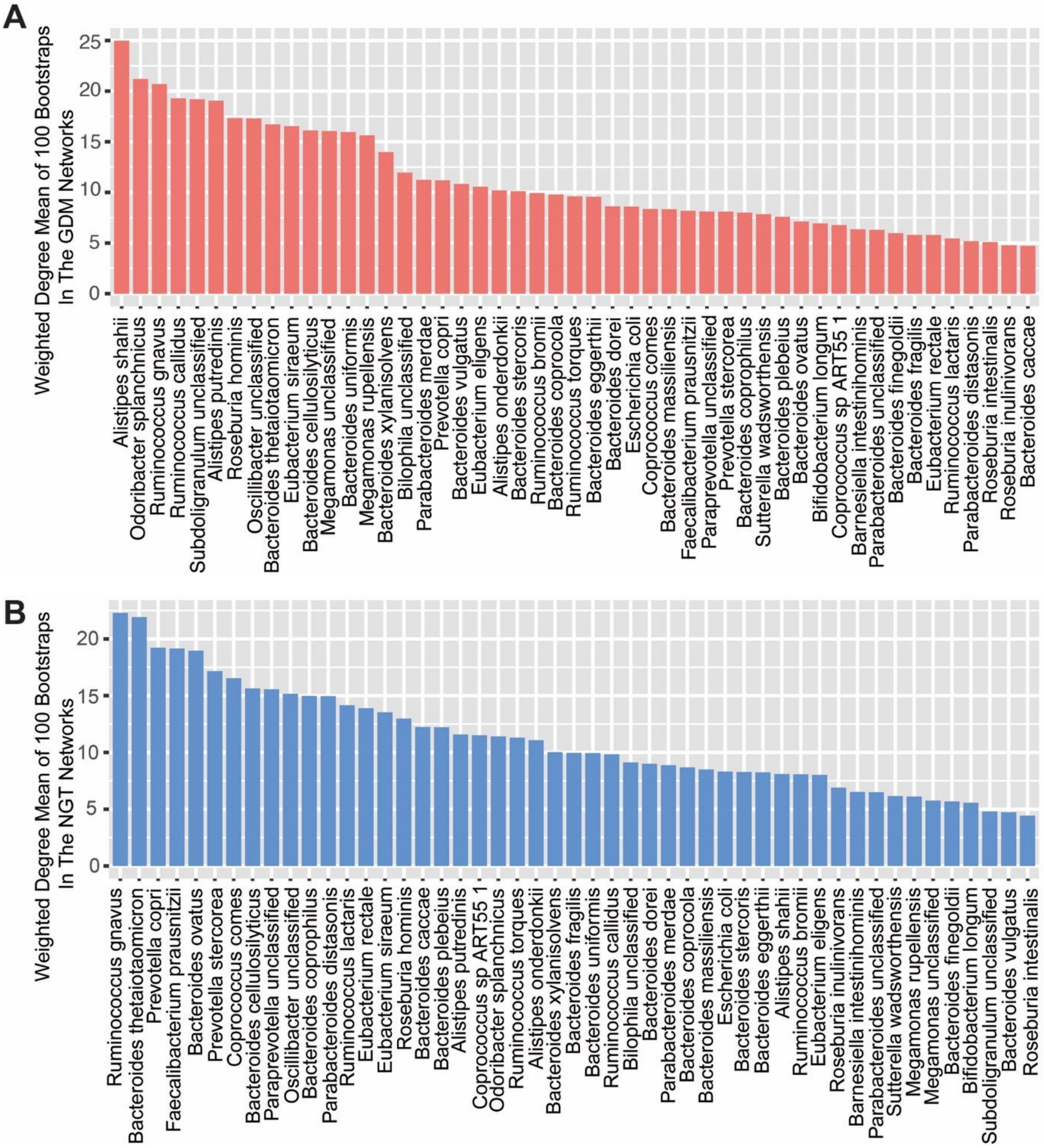

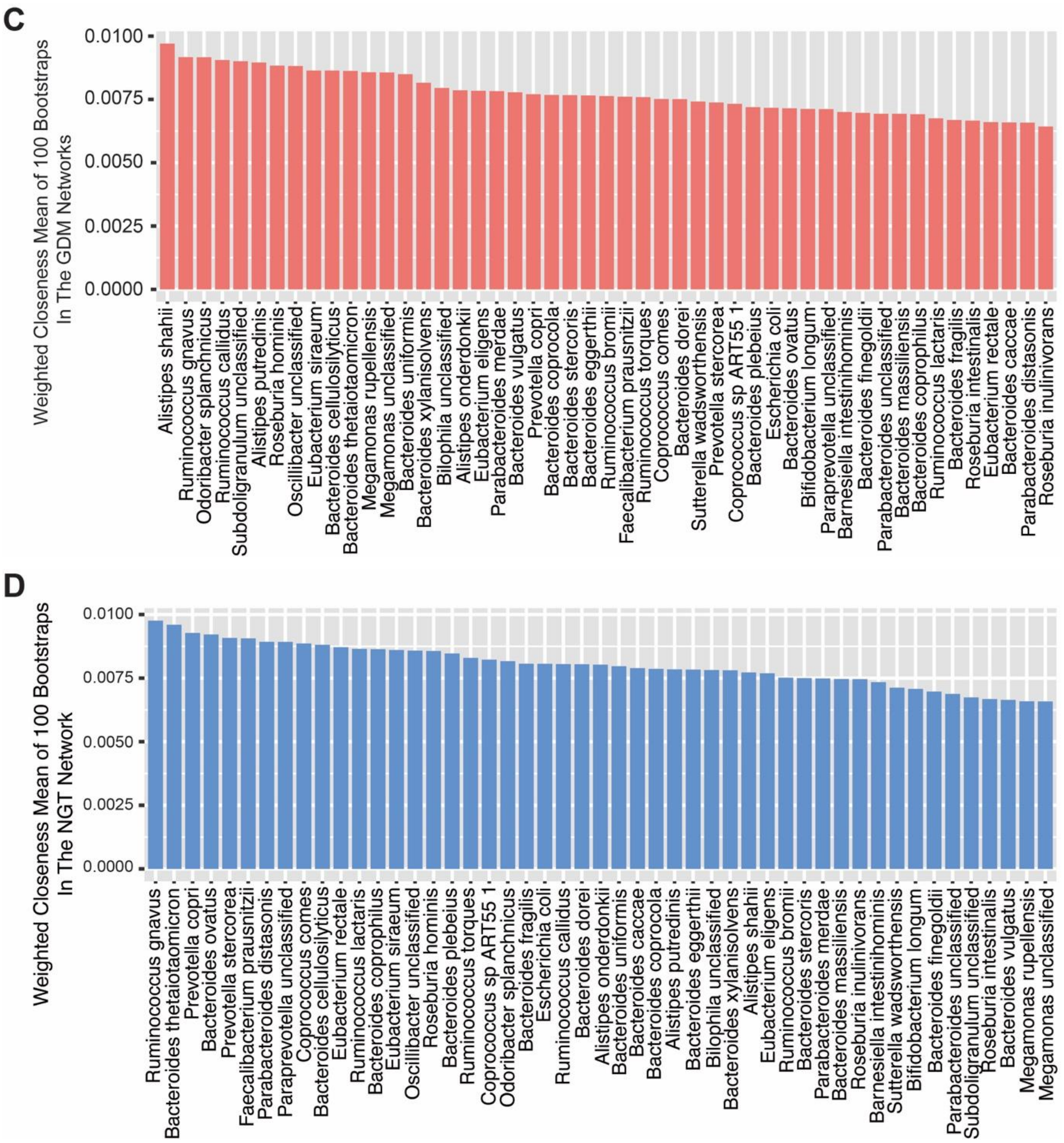

Supplementary Fig. 5. The gut microbial species in the sub-network structure

Means of the weighted degree of 100 sub-networks from GDM (A) and NGT (B). Means of the weighted closeness of 100 sub-networks from GDM (C) and NGT (D). The sub-network was constructed in randomly selected 30 samples from both GDM and NGT group. This procedure was repeated 100 times then we got 100 sub-networks from each group to calculate the average of weighted degree and weighted closeness of gut microbial species.

References in *Supplementary Materials*

1. Lee D, Lee Y, Pawitan Y, Lee W. Sparse partial least-squares regression for high-throughput survival data analysis. Stat Med 2013; 32:5340-52.
2. Oksanen J, Blanchet FG, Kindt R, Legendre P, Minchin PR, O'hara R, et al. Package 'vegan'. Community ecology package, version 2013; 2:1-295.
3. Rivera-Pinto J, Egozcue JJ, Pawlowsky-Glahn V, Paredes R, Noguera-Julian M, Calle ML. Balances: a New Perspective for Microbiome Analysis. mSystems 2018; 3.
4. Csardi G, Nepusz T. The Igraph Software Package for Complex Network Research. InterJournal 2005; Complex Systems:1695.
5. Chun H, Keles S. Sparse partial least squares regression for simultaneous dimension reduction and variable selection. J R Stat Soc Series B Stat Methodol 2010; 72:3-25.
6. Aggio RB, Ruggiero K, Villas-Boas SG. Pathway Activity Profiling (PAPi): from the metabolite profile to the metabolic pathway activity. Bioinformatics 2010; 26:2969-76.
7. Lundberg SM, Lee S-I. A unified approach to interpreting model predictions. Advances in neural information processing systems 2017; 30.
